# The Financial and Environmental Cost of Anaesthetic Emergency Drugs: Comparing Ampoules With Prefilled Syringes

**DOI:** 10.7759/cureus.105373

**Published:** 2026-03-17

**Authors:** Edward A Parkinson, Eleanor Plews, Chinthaka Hewavitharane, Grace Catchpole, Rose-Anne Nunoo, Himanshu Arora, Champa Chapman

**Affiliations:** 1 Anaesthesia, East Kent Hospitals University NHS Foundation Trust, Ashford, GBR; 2 Anaesthesia, East Kent Hospitals University NHS Foundation Trust, Margate, GBR; 3 Acute Care, East Kent Hospitals University NHS Foundation Trust, Ashford, GBR

**Keywords:** anaesthesia, carbon footprint, cost-benefit analysis, emergency drugs, environmental sustainability, pharmaceutical waste, prefilled syringes

## Abstract

Introduction

Anaesthetic emergency drugs are essential for safe practice; however, when prepared from ampoules, they are frequently wasted when unused. Prefilled syringes (PFS) may reduce waste as they can be opened at the point of use. Additional benefits may include reducing medication errors and saving clinician time, although higher initial purchasing costs have been a barrier to universal adoption across the NHS. We undertook two quality improvement cycles to quantify the use and waste of anaesthetic emergency drugs before and after transitioning from ampoules to PFS for metaraminol, ephedrine, and atropine, measuring cost, waste generated, and environmental impact.

Methods

This was a prospective, observational study conducted across nine operating theatres at Queen Elizabeth the Queen Mother Hospital, Margate, United Kingdom, quantifying the use and waste of anaesthetic emergency drugs. Surveys were distributed to each theatre and completed by anaesthetic teams through February 2024 for cycle 1 and February 2025 for cycle 2. Cycle 1 collected baseline data on emergency drug use and waste for metaraminol, ephedrine, atropine, propofol, and glycopyrrolate. We repeated this for cycle 2 after transitioning to PFS for metaraminol, ephedrine, and atropine. Annual use and waste of emergency drugs were estimated with costs of ampoules, PFS, and equipment, including syringes, sharps bin disposal, and waste incineration provided by hospital pharmacy and facilities teams. Kilograms of carbon dioxide equivalent (kgCO2e) were estimated using emission and conversion factors based on NHS financial expenditure and incineration of the generated waste weight according to the Sustainability in Quality Improvement guidelines.

Results

Cycle 1 returned 28 surveys describing 87 surgical cases. Of 208 emergency drugs prepared from glass ampoules, 100 (48.1%) were wasted, costing an estimated £23,348 a year, equivalent to £2,594/theatre/year and producing 196.8 kg of waste. This corresponded to an annual production of an estimated 3,372 kgCO2e, equivalent to 52 flights from London to Paris. Cycle 2 returned 87 surveys comprising 270 surgical cases. Wasted drugs reduced to 15/159 (9.4%), with the cost of waste falling by 91.1% to £231/theatre/year. Waste weight reduced by 93.0% to 13.8 kg, with associated emissions falling by 91.3% to 294 kgCO2e. Replacing metaraminol, ephedrine, and atropine ampoules with PFS yielded an estimated annual cost saving of £14,042 (31.6%), equivalent to £1,560/theatre/year.

Conclusions

Replacing metaraminol, ephedrine, and atropine ampoules with PFS was associated with substantial reductions in anaesthetic emergency drug costs, medication waste, and the environmental impact associated with pharmaceutical disposal. These reductions were accompanied by changes in drug preparation practices in the theatre. Limitations include self-reported survey data, the use of NHS-specific conversion factors for kgCO2e estimation, and not assessing the financial or human costs of potential medication errors. Generalisability may be limited by the single-centre study design. PFS may represent a useful strategy to reduce waste, costs, and environmental impact while supporting medication safety in anaesthetic practice.

## Introduction

Anaesthetic emergency drugs are essential for safe practice; however, when prepared from glass ampoules and unused, are frequently discarded at the end of surgical lists as waste. Prefilled syringes (PFS) are ‘ready to administer’ medications which may reduce pharmaceutical waste as they can be opened at the point of use. Additional benefits include reducing medication errors, improved protection against tampering, and saving clinician time, although higher initial purchasing costs have been a barrier to universal adoption across NHS trusts [1,2].

The NHS has committed to achieving net-zero by 2040, and improving the sustainability of anaesthetic practice has become an increasing priority [3,4]. Adoption of PFS has been further supported by national guidance produced by a working group including the Royal College of Anaesthetists and the Association of Anaesthetists, endorsed by the Royal Pharmaceutical Society, first published in 2020 and updated in January 2026 [5]. Notably, national tariffs indicate a substantial reduction in the price of ephedrine PFS alongside an increase in the cost of ephedrine ampoules, suggesting little advantage to procuring ephedrine ampoules for intravenous use [6].

To our knowledge, no studies comparing the costs of ampoules and PFS within the NHS have previously been published. Two NHS quality improvement projects, however, have explored environmental benefits. One project described an 84.1% reduction in wasted syringes and 86% reduction in kilograms of carbon dioxide equivalent (kgCO2e), while the second project found a reduction in emergency drug waste from 64% to 40% [7,8]. Studies in other healthcare settings have quantified significant emergency drug waste, with some identifying PFS as a cost-saving strategy [2,9-12]. The July 2022 update to the 2020 commitment to deliver a net zero NHS deemed medicines as responsible for 25% of emissions, with waste processes identified as areas for optimisation [3].

PFS advantages are not limited only to waste. Medication errors have previously been reported as 17 times less likely using PFS while additionally reducing preparation time [13]. From a human factors perspective, removing steps in drug preparation may reduce human error, provide more accurate drug concentrations, and reduce cognitive burden in emergencies [14,15]. PFS has also been associated with a reduction in catheter-related bloodstream infections [16].

We hypothesised that transitioning from ampoules to PFS for metaraminol, ephedrine, and atropine would significantly reduce pharmaceutical waste and the associated carbon footprint, and any increased acquisition costs would be offset by the generation of less waste. The aims of this project were therefore to estimate the use and waste of anaesthetic emergency drugs, quantify the associated carbon footprint, and compare the financial costs of ampoules and PFS.

The Cycle 1 results of this study were presented, in part, at the Spring 2024 *British Journal of Anaesthesia* (BJA) Research Forum on May 17, 2024, and at the Association of Anaesthetists (AoA) Annual Congress on September 11, 2024, with the abstract published in the BJA [17]. Cycle 2 results of this study were presented, in part, at the Spring 2025 BJA Research Forum on May 16, 2025, and at the AoA Annual Congress on September 18, 2025, with the abstract published in the BJA [18].

## Materials and methods

We designed a prospective, observational study informed by the Sustainability in Quality Improvement (SusQI) and SQUIRE (Standards for QUality Improvement Reporting Excellence) 2.0 guidelines [19,20]. The study was conducted at Queen Elizabeth the Queen Mother Hospital, Margate, United Kingdom. Institutional Review Board/Institutional Ethics Committee approval was not required. No patient-identifiable data was collected, and patients were not randomised or received treatments that deviated from standard care. The project was registered locally with the Improvement and Transformation Team.

Our acute, district general hospital contains nine non-obstetric theatres undertaking approximately 12,000 surgical cases per year. All elective and emergency operating lists in the general theatres during February 2024 for cycle 1 and February 2025 for cycle 2 were eligible for inclusion. Operating lists in obstetric theatres were excluded. Convenience sampling, using a paper-based data collection proforma, was distributed to each theatre to be completed contemporaneously by anaesthetic teams, establishing the baseline use and waste of anaesthetic emergency drugs. Data capture relied on voluntary self-reporting by clinical teams, with consent implied by survey completion. Further validation of survey compliance was not performed. Additional data were collected to describe the surgical specialty, the number of patients, and the American Society of Anesthesiologists (ASA) status. Emergency drugs included metaraminol, ephedrine, atropine, emergency propofol, and glycopyrrolate. We additionally surveyed wasted medical equipment by recording the number of syringes and filter needles opened for rapid access, alongside unopened ampoules. It should be noted that only drugs that were used or wasted were counted. Unused PFS were not in the total count.

Data collection was repeated in February 2025 for cycle 2 following the transition from glass ampoules to PFS for metaraminol, ephedrine, and atropine, and after a local recommendation not to routinely open glycopyrrolate glass ampoules unless there was a specific clinical concern, instead having unopened ampoules stored readily alongside empty syringes in the emergency drug tray. Results are reported as counts and percentages, with annualised use and waste of emergency drugs and medical equipment estimated, extrapolated from the one-month data collection cycle for 12,000 annual cases. Costs of ampoules, PFS, equipment, including syringes, sharps bin disposal, and waste incineration, were provided by hospital pharmacy, suppliers, and facilities teams. kgCO2e were estimated using emission factors based on NHS financial expenditure in addition to a conversion factor for the incineration of the generated waste weight according to the SusQI guidelines [19]. Everyday comparisons for road and air miles were made using government conversion factors [21].

We did not adjust for potential confounders, including case mix, surgical specialty, staffing grade, or patient acuity, as the study was designed to be descriptive. On this basis, no statistical tests were performed, and confidence intervals were not calculated as drug waste was measured per list rather than per patient. In order to improve generalisability, we repeated the financial analysis using national costs from the February 2024 NHS Business Service Authority (NHSBSA) drug tariff or, if unlisted, the cheapest medicinal form of the same concentration available in the British National Formulary [6,22].

## Results

The unit cost of ampoules and PFS for local and national tariffs are presented in Table 1.

**Table 1:** The cost per unit of ampoules, medical equipment used to administer them, and prefilled syringes *Drug Tariff February 2024 [6]; Joint Formulary Committee, British National Formulary [22] NHSBSA: NHS Business Service Authority; BNF: British National Formulary

Item	Concentration, volume	Cost per unit, local tariff (£)	Cost per unit, NHSBSA/BNF (£)*
Ampoules			
Metaraminol	0.5mg/ml, 5ml	2.805	8.28
Ephedrine	30mg/ml, 1ml	4.5	17.581
Propofol	10mg/ml, 20ml	0.46	0.96
Atropine	600mcg/ml, 1ml	2.11	1.171
Glycopyrrolate	200mcg/ml, 3ml	2.5	2.112
0.9% NaCl	0.9%, 10ml	0.499	0.424
Medical equipment			
3ml syringe	-	0.0242	-
5ml syringe	-	0.0275	-
10ml syringe	-	0.0592	-
20ml syringe	-	0.1012	-
Filter needles	-	0.0389	-
Prefilled syringes			
Metaraminol	0.5mg/ml, 5ml	5.5	9.5
Ephedrine	3mg/ml, 10ml	4.4	9.5
Atropine	500mcg/ml, 5ml	5	13

Cycle 1 during February 2024 returned 28 surveys comprising 87 surgical cases. In 12 (42.9%) of the 28 surveys, at least one patient had an ASA grade of ≥3. When prepared from glass ampoules, 100 (48.1%) of the total 208 anaesthetic emergency drugs were wasted, estimated at 966 emergency drugs/theatre/year. The percentage utilisation of each drug was: metaraminol 26/36 (72.2%), ephedrine 22/36 (61.1%), emergency propofol 18/32 (56.3%), atropine 2/16 (12.5%), and glycopyrrolate 0/11 (0%). Local tariffs estimated the annual cost of this waste as £23,348 (equivalent to £2,594/theatre/year), including waste disposal and incineration. Wasted ephedrine contributed the greatest financial burden, followed by atropine. The annual production of waste was estimated to be 196.8 kg, comprising 77.8 kg plastic, 53.9 kg drug solution, 32.2 kg paper or cardboard, 29.8 kg glass, and 3.1 kg metal. The associated kgCO2e produced from this waste was estimated at 3,372 kgCO2e annually (equivalent to 375 kgCO2e/theatre/year). Using cycle 1 data, we modelled that replacing metaraminol, ephedrine, and atropine ampoules with PFS would yield a 22.4% cost saving of £9,933 a year, with national drug tariffs predicting a greater but proportionate saving of 21.5% at £18,164.

Cycle 2 during February 2025 and following the transition from glass ampoules to PFS for metaraminol, ephedrine, and atropine, returned 87 surveys comprising 270 cases. PFS were available in 82 of the 87 (94.3%) surveys, with a small proportion of glass ampoules still in circulation. In 50 (56.3%) surveys, at least one patient had an ASA grade of ≥3. We found an 80.5% reduction in emergency drug waste from 100/208 (48.1%) to 15/159 (9.4%), with the extrapolated annual cost of waste reducing by 91.1% from £23,348 to £2,080 (equivalent from £2,594/theatre/year to £231/theatre/year). Utilisation of emergency drugs increased following the transition to PFS: metaraminol 63/66 (95.5% from 72.2%), ephedrine 69/75 (92.0% from 61.1%), and atropine 3/3 (100% from 12.5%). The weight of generated waste reduced by 93.0% from 196.8 kg to 13.8 kg, contributing to a 91.3% reduction in kgCO2e produced from waste from 3,372 to 294 kgCO2e (equivalent from 375 kgCO2e/theatre/year to 32 kgCO2e/theatre/year) (Table 2).

**Table 2:** Estimated annual kgCO2e from anaesthetic emergency drugs, comparing ampoules with prefilled syringes *Sustainability in Quality Improvement (SusQI) [19] kgCO2e: kilograms of carbon dioxide equivalent

Waste type	Cycle 1 quantity	Cycle 2 quantity	Conversion factor [19]*	Cycle 1 kgCO2e	Cycle 2 kgCO2e
Pharmaceutical waste cost	£21,832.44	£1,971.24	0.128 per NHS £	2,795	252
Medical equipment waste cost	£795.78	£58.10	0.46 per NHS £	366	27
Weight of waste for incineration	196.8kg	13.8kg	1.074 per kg	211	15
Total				3,372 kgCO2e	294 kgCO2e

**Figure 1:**
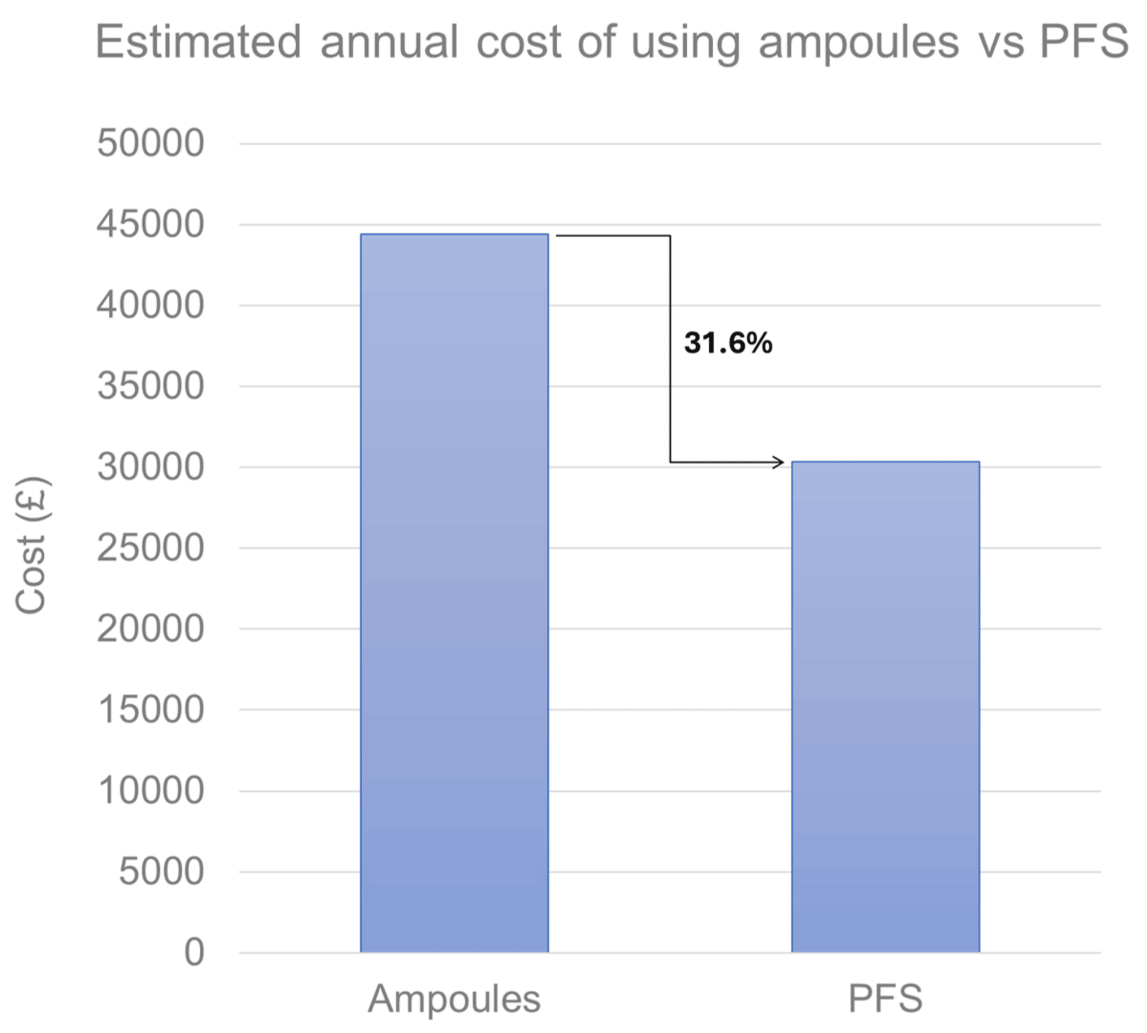
The financial cost of ampoules versus prefilled syringes for metaraminol, ephedrine, and atropine, using local drug tariffs PFS: prefilled syringe

Using local drug tariffs, the total cost of acquisition, use, and waste of PFS compared with glass ampoules specifically for metaraminol, ephedrine, and atropine showed an estimated 31.6% reduction from £44,386 to £30,344 annually, saving £1,560/theatre/year (Figure 1). National NHSBSA tariffs suggested a greater reduction of 51.2%, or £7,573/theatre/year.

## Discussion

Of the total prepared anaesthetic emergency drugs, 48.1% were wasted in cycle 1, which is comparable with existing literature [7-9,11,12]. This was reduced significantly to 9.4% following the transition to PFS for three of the five assessed emergency drugs in cycle 2. The observed 31.6% cost saving of £1,560/theatre/year was greater than the 22.4% predicted from cycle 1. This additional saving reflected a concurrent change in practice. For example, following local recommendation, drugs available only as ampoules, such as glycopyrrolate, were less routinely prepared; instead, an unopened ampoule was stored alongside an empty syringe in the emergency drug tray for rapid access if required. Additionally, 9/66 (13.6%), approximately 15% of all used metaraminol in cycle 2, was prepared from glass ampoules still in circulation, which are currently less expensive than their PFS counterparts. Nevertheless, assuming the complete removal of ampoules in circulation, the cost reduction remains significant at 29.1% (£1,435/theatre/year) using local tariffs.

We appropriately encourage the preparation of any emergency drug for higher-risk patients, including those with higher ASA status, those with relevant comorbidities, and where anaesthetists earlier in training may prefer more interventions to be immediately available. These circumstances may necessitate increased preparation of emergency drugs with a corresponding increase in the potential for waste; however, this is justified if enhancing patient safety, which must remain paramount. There may also be psychological factors, not measured in our study, influencing clinician behaviour. Ampoule-based emergency drugs are often prepared in advance which may lower the threshold for their administration if they would otherwise be discarded as waste. In contrast, PFS remain sealed and require an additional step before use, potentially creating a small practical barrier encouraging their use only when clearly indicated.

National drug tariffs were used for a second cost-analysis to improve generalisability between trusts. While suggesting greater savings, we believe it is more reasonable that other NHS trusts would secure procurement costs comparable to our trust.

Following the reduction in waste produced, we quantified a corresponding reduction in kgCO2e. In cycle 1, 3,372 kgCO2e was estimated to be produced from waste annually, equivalent to driving an average petrol car 12,783 miles, or 52 flights from London to Paris [21].

Limitations include self-reported survey data, use of NHS-specific conversion factors for kgCO2e estimation rather than primary lifecycle analysis, and not assessing the financial and human costs of potential medication errors. Further studies across multiple centres are required to provide additional validation of our findings. Although the kgCO2e associated with waste was reduced, we were unable to obtain comparative data for kgCO2e associated with the manufacturing of ampoules vs PFS. We also did not assess theatre or clinician efficiency, where PFS reduces drug preparation time with improved downstream effects. Generalisability of the magnitude of observed cost savings and waste reduction may be limited by our single-centre study design; however, waste reduction is likely to be reproducible and therefore translatable to other centres implementing similar changes.

Increased market availability and production of ephedrine PFS appear to have reduced unit costs below those of ampoules, further supporting the transition to PFS [6]. As manufacturers expand PFS production for other emergency drugs and medicines, similar cost reductions may occur over time. Patient safety, financial, and efficiency benefits may also extend to non-theatre settings, particularly relevant for critical care, prehospital, and emergency medicine.

## Conclusions

Almost half of all prepared ampoule-based anaesthetic emergency drugs were wasted. Introducing PFS for metaraminol, ephedrine, and atropine, significantly reduced overall emergency drug waste and its associated CO2 burden. PFS were more cost-effective than ampoules, primarily due to the generation of less waste. Waste reduction was achieved through higher utilisation of PFS and a change in practice whereby ampoule-based emergency drugs, including glycopyrrolate, were less routinely prepared. Limitations of this study include the reliance on self-reported survey data and the single-centre design, which may limit generalisability; multicentre studies are therefore required to validate these findings. Nevertheless, our findings suggest PFS represent a cost-effective and practical strategy delivering financial, environmental, and safety benefits for patients, the NHS, and the planet.

## Figures and Tables

**Figure 2:**
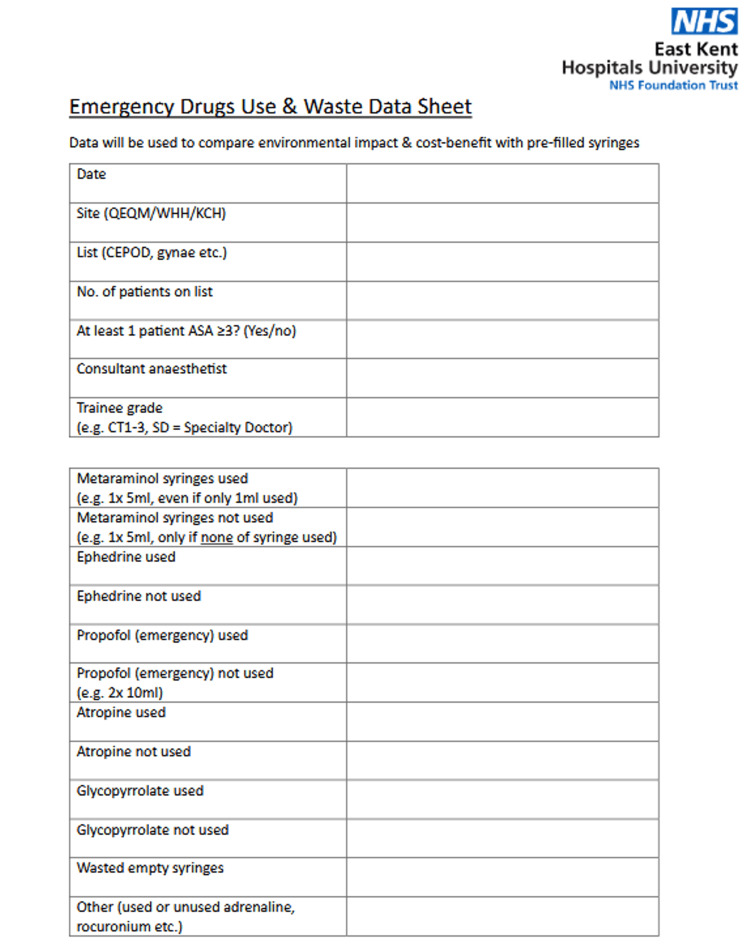
Cycle 1 data collection sheet

**Figure 3:**
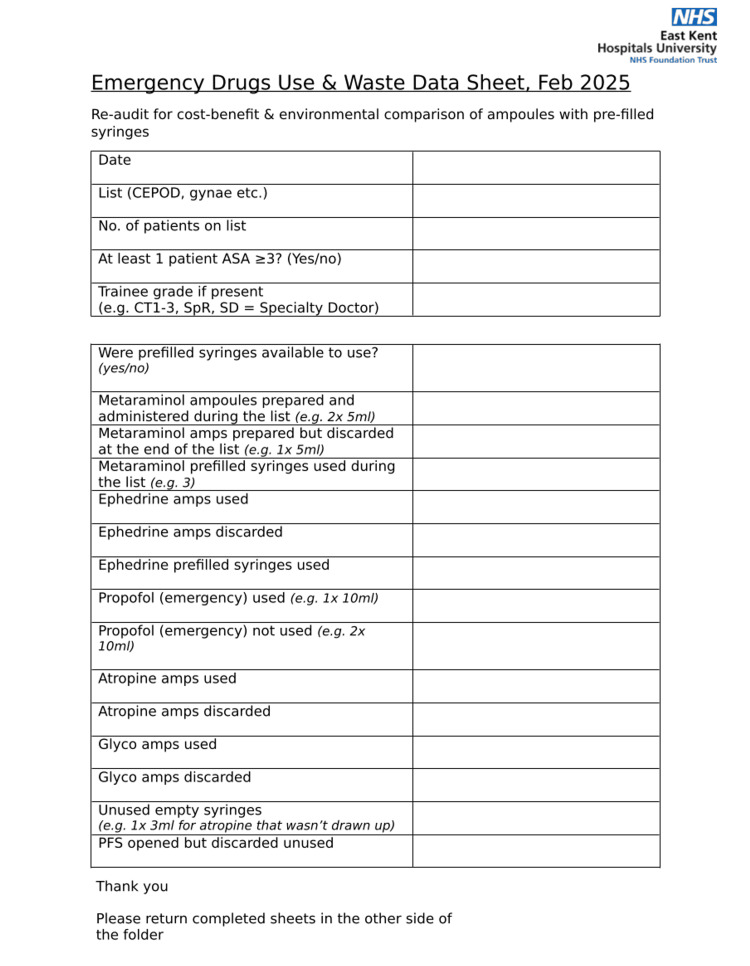
Cycle 2 data collection sheet, adapted from cycle 1

## Appendices

Cycle 1

 Cycle 2
